# Therapist versus Machine—Immediate Effects of Manual versus Mechanical Lymphatic Drainage in Patients with Secondary Lymphedema

**DOI:** 10.3390/jcm13051277

**Published:** 2024-02-23

**Authors:** Daniel Schiltz, Dominik Eibl, Karolina Mueller, Niklas Biermann, Lukas Prantl, Christian Dirk Taeger

**Affiliations:** 1 Department of Plastic, Hand- and Reconstructive Surgery, University Hospital Regensburg, 93053 Regensburg, Germanyniklas.biermann@ukr.de (N.B.); lukas.prantl@ukr.de (L.P.); 2 Department of Plastic and Aesthetic Surgery, Hand Surgery, Helios Hospital Emil von Behring, 14165 Berlin, Germany; 3 Center for Clinical Studies, University Hospital Regensburg, 93053 Regensburg, Germany; karolina.mueller@ukr.de

**Keywords:** lymphatic drainage, intermittent pneumatic compression, manual lymphatic drainage, lymphedema, lipedema

## Abstract

**Background**: Complex decongestive therapy (CDT) is the standard and basic therapy for lymphedema. The central component of CDT is manual lymphatic drainage (MLD). In addition to CDT, other measures such as intermittent pneumatic compression therapy (IPCT) (active compression machine therapy) are available. In this prospective research study, the objective and subjective effects of MLD and IPCT on lymphedema of the lower extremity were investigated and both therapies were directly compared. Furthermore, the patients’ body mass index (BMI) and stage of lymphedema were tested for their effect on the respective therapy. **Methods**: Patients participating in the study received both therapies (MLD and IPCT) on the same lymphedema-affected limb at an interval of two days. The objective volumetric therapy effect was measured by the digital volume measurement of the affected limb. The subjective effects of the therapies were measured using two specially designed questionnaires. **Results**: A total of 40 patients were included in the study. There was no significant difference in the volume differences between the interventions, BMI categories, lymphedema, or treatment order regarding the immediate and two-day effect. **Conclusions**: No significant difference was found in the subjective or objective therapy efficacy of the two methods. Intermittent pneumatic compression therapy is considered a comparable therapeutic procedure when properly indicated.

## 1. Introduction

Chronic lymphedema is an inflammatory lymphostatic disease of the interstitium, resulting from primary (congenital) or secondary (acquired) damage to the lymphatic drainage system. It is characterized by the mechanical insufficiency of the lymphatic vascular system, i.e., the initial lymphatic vessels, precollectors, lymphatic collectors, trunci, and/or lymph nodes. This results in the decreased drainage capacity of the lymphatic system. Consequently, a mismatch of the lymphatic load and drainage capacity leads to edema. This process cumulates in the trophic disruption of the surrounding organs, the proliferation of parenchyma, and in connective tissue (fibrosis and sclerosis) and adipose tissue in the interstitium [1,2,3]. The staging is based on clinical criteria. Depending on the literature, 3 or 4 stages are distinguished, whereby the classification that includes the latency stage (4 stages) has become widely accepted [1,4]. Often, lymphedema triggers multiple symptoms and pathologies. Common general sequelae of lymphedema are pain, itching, a feeling of heaviness and fatigue, skin changes and infections (29%) [5]. About half of all lymphedema patients suffer from these symptoms [5]. Many patients feel that lymphedema massively restricts their personal activities [6,7,8].

The data available on the epidemiological parameters are still not very reliable [4]. Worldwide, more than 200 million people are believed to suffer from lymphedema [9]. Pathophysiological causes vary widely among industrialized, emerging, and developing countries [4]. In industrialized nations, the most common cause of secondary lymphedema is malignancy and the consequences of its treatment (surgery, radiotherapy and chemotherapy), accounting for approximately 15.5–50.8% of cases [5,9].

Complex decongestive therapy (CDT) is the standard and basic therapy for lymphedema [4,10,11]. An important component of CDT is manual lymphatic drainage (MLD). Pressure and stretching on the cutis and subcutis result in an increase of lymph flow in the lymphatic collectors [4,10,12,13,14]. In addition to CDT, other measures such as intermittent pneumatic compression therapy (IPCT) are available. IPCT is an approved [15,16,17,18,19] therapy (especially in inpatient settings [20]) that, despite its potential as a home and continuous therapy, seems to be less established. There is an unsatisfactory situation in the study of the therapy of chronic lymphedema in general, and in manual lymphatic drainage and intermittent pneumatic compression in particular [21,22]. Within the guidelines, IPC therapy is only recommended as an additive therapy to CDT [4,23]. To date, it is unclear how strong the decongestive effect and patient acceptance of IPCT are in direct comparison to MLD. In this prospective research study, the objective and subjective effects of MLD and IPCT on lymphedema of the lower extremity are investigated and both therapies are directly compared. Furthermore, the patients’ body mass index (BMI) and stage of lymphedema were tested for their effect on the respective therapy.

## 2. Materials and Methods

**Figure 1:**
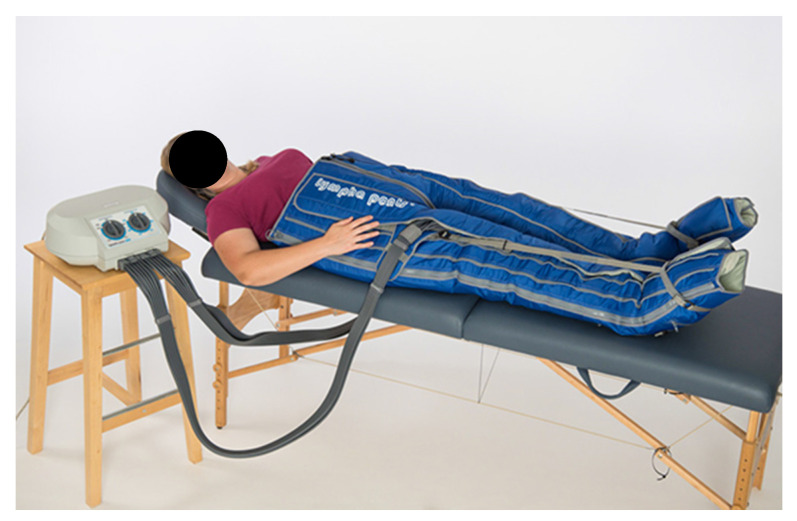
Device used for IPCT in this study. (Lympha Press Mini—12-step system, Villa-Sana, Weiboldshausen, Germany).

**Figure 2:**
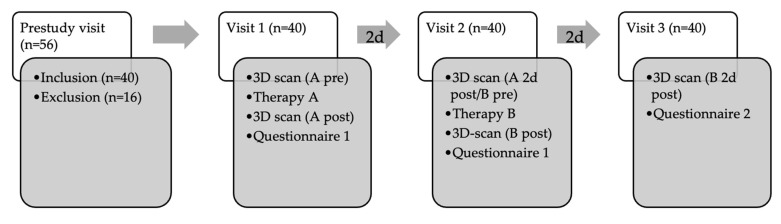
Study process. (n = number of patients; pre = immediately before therapy; post = immediately after therapy; 2d = two days).

This prospective, cross-over study was approved by the Ethics Committees of the University of Regensburg (reference number:19-1587-101; date of approval: 13 November 2019). Patients gave their written informed consent. The inclusion criteria were secondary stage I-III lymphedema of the lower extremities. The exclusion criteria were any other lymphedema (e.g., primary lymphedema), infection (erysipelas, phlegmon or chronic wound), cardiac insufficiency, active malignant disease, epilepsy (due to the flashing light emitted during the scanning process), increased photosensitivity, and patient refusal. All included patients received a guideline-based conservative therapy before inclusion. Therapy was stopped 7 days before the patients underwent the first study therapy. Patients participating in the study received two cycles of therapy on the same lymphedema-affected limb at an interval of two days. One therapy cycle consisted of MLD and the other of IPCT. The order was randomized (blinded random assignment). The sequence of therapies was randomized with a washout phase of two days between therapies. MLD was performed by therapists (in total 8) who were trained and certified in manual lymphatic drainage. Before MLD, the patient’s proximal lymphatic pathways were cleared. Figure 1 shows the device used for IPCT. The therapy time for each session (MLD and IPC) was 45 min. The device works with an associated stocking or pants. Pants were solely used in this study (Figure 1). The patient’s proximal lymphatic pathways were not cleared prior to IPCT (as this would not be the case in everyday life). The immediate success of MLD and IPCT on the lower extremity was assessed both objectively and subjectively. The study process is shown in Figure 2.

### 2.1. Objective Measurements (3D Volumetry)

The objective volumetric therapy effect was measured by digitally measuring the volume of the affected limb (3D volumetry). Digital volumetric measurements based on 3D scans have already proven to be a valid method for the volumetric assessment of the leg [24]. Scans were performed before the therapy, immediately after the therapy, and two days after the therapy (resulting in a total of five scans per patient).

### 2.2. Subjective Measurements

The subjective effect of the therapies was measured by two specially designed questionnaires (not validated) that were filled in immediately after each therapy (questionnaire 1) and two days after the last therapy session (questionnaire 2).

#### 2.2.1. Questionnaire 1

This questionnaire was filled in immediately after each therapy session and refers to the therapy that has just been completed. It contains 6 items. Item 1 asks about the pain during the therapy with a visual analog scale. Item 2 inquires about the occurrence of paresthesia using a 5-point scale. In Item 3, this paresthesia can be specified. Item 4 asks whether the subjects found the therapy pleasant and is based on a 5-point Likert-type scale. Item 5 asks whether the therapy was perceived as effective, again using a 5-point Likert-type response scale. Item 6 asks whether the study subjects would recommend the intervention just received to other patients (yes/no).

#### 2.2.2. Questionnaire 2

This questionnaire was filled in during the follow-up period, two days after the last therapy session. It contains 4 items, refers to both therapy techniques and focuses on the long-term effects. Item 1 asks about the subjective long-term effect (a 5-point scale between <12 h to >48 h) of MLD. Item 2 asks the same question about the subjective long-term effect of IPCT. Item 3 asks subjects about their preferred intervention (MLD or IPCT). Item 4 asks the subjects whether they could imagine having the therapy performed exclusively by the automatic device (IPCT) (yes/no).

### 2.3. Statistics

The statistical analysis was performed using the IBM^®^ SPSS^®^ Statistics program (version 25, 64-bit version). Data are presented descriptively as absolute and relative frequencies for categorical variables, as the mean (±standard deviation (SD)) for normally distributed data or as the median (interquartile range (IQR) for non-normally distributed continuous data. To compare the immediate effects (volume difference in mL between pre and immediately post treatment) and two-day effects (volume difference in mL between pre and two days post treatment) of MLD and IPCT, two separate linear mixed models were computed. These included the co-variables BMI (obese ≥ 30 vs. non-obese < 30), lymphedema stage (I vs. II), treatment order, and baseline volume. Adjusted means and corresponding 95% confidence intervals (CI) are presented. Wilcoxon tests were used to compare the subjective effects (effectiveness, long-term effectiveness in hours, pain, paresthesia, and pleasantness/unpleasantness) of the therapies. Fisher’s exact tests were used to compare the preferred therapy between age groups (<67 years vs. ≥67 years) and employment status (employed vs. unemployed). The level of significance was set at *p* ≤ 0.05 for all tests. Data analyses were exploratory in nature. Therefore, no adjustments were made for multiple testing.

### 2.4. Patient Recruitment

In total, 56 patients were seen in the pre-study visit. Patients were recruited from the plastic surgery consultation at the University Hospital Regensburg, by lymph therapists in private practice and by general practitioners. Following screening, 16 patients were excluded (n = 11 with misdiagnosis; n = 3 with primary lymphedema; n = 2 due to patient refusal). A total of 40 patients were finally included in the study.

## 3. Results

Of the 40 patients included in the study, 32 were female (80%) and 8 were male (20%). The mean age was 59.4 (±14.0) years. The mean body mass index (BMI) was 31.9 (±7.4). A total of 19 patients suffered from lymphedema stage I and 21 were classified as stage II. In 33% of patients (n = 13), lymphedema occurred after cancer therapy.

### 3.1. Objective Measurements (3D Volumetry)

#### 3.1.1. Immediate Effect

**Figure 3:**
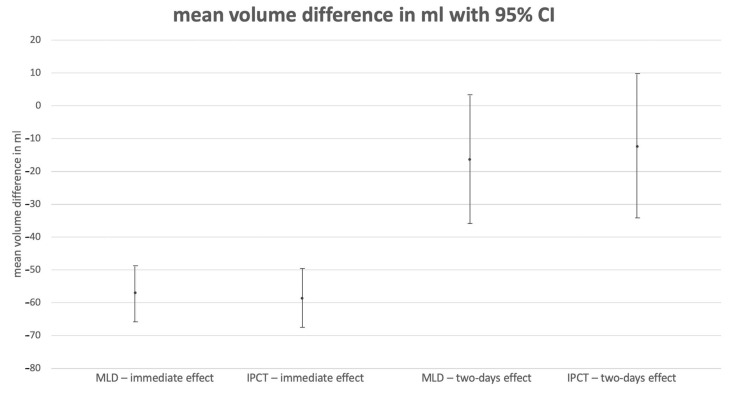
Results of the objective (3D volumetric) measurements of MLD and IPCT at both measurement points (immediately after therapy and two days after therapy) in mL. (MLD = manual lymphatic drainage; IPCT = intermittent pneumatic compression therapy; CI = confidence interval).

There was no significant difference in the volume difference between the interventions (mean difference = 1.61 mL, 95% CI = −9.99/13.22, *p* = 0.780, Figure 1), BMI categories (mean difference = 11.71 mL, 95% CI = −3.88/27.29, *p* = 0.137), lymphedema stage (mean difference = 7.66 mL, 95% CI = −5.45/20.76, *p* = 0.245), and treatment order (mean difference = 9.37 mL, 95% CI = −4.20/22.95, *p* = 0.171). Results are shown in Table 1 and Figure 3.

#### 3.1.2. Effect after Two Days

There was no significant difference after two days in the volume difference between interventions (mean difference = −4.04 mL, 95% CI = −37.91/29.84, *p* = 0.811, Figure 1), BMI categories (mean difference = 5.94 mL, 95% CI = −21.17/33.04, *p* = 0.660), lymphedema stage (mean difference = 16.44 mL, 95% CI = −6.33/39.20, *p* = 0.152), and treatment order (mean difference = 4.10 mL, 95% CI = −19.49/27.68, *p* = 0.727). Results are shown in Table 1 and Figure 3.

### 3.2. Subjective Measurements

None of the participants perceived the interventions as having “little” effectiveness or being “not at all” effective. Therefore, these response options are not presented in the table for better readability. The subjective effectiveness of both therapy options was reported in 15/40 (37.5%) patients. The effectiveness of IPCT was considered greater than that of MLD by 10/40 (25.0%) patients. Conversely, patients perceived the effectiveness of MLD to be higher than the effectiveness of IPCT in 15/40 (37.5%) cases. The Wilcoxon test showed no significant difference in the subjective effectiveness of the therapies (*p* = 0.257). Results are shown in Table 2.

A total of 17/40 (42.5%) patients estimated the long-term effect of both interventions to be the same. The patients who rated the long-term effect of IPCT as more long-lasting compared to manual lymphatic drainage amounted to 14/40 (35.0%) cases, while 9/40 (22.5%) perceived the effect of MLD to be more long-lasting. There was no significant difference in the subjective long-term effectiveness of the therapies (*p* = 0.117). Results are shown in Table 3.

The subjective pain experienced during the respective therapy application was indicated by the patients directly after the intervention on a visual analog scale (0 = no pain; 10 = very severe pain). The Wilcoxon test showed no significant difference in the patients’ perception of pain between MLD (median = 0.0, IQR = 0.0/0.5) and IPCT (median = 0.0, IQR = 0.0/0.5) (*p* = 0.571).

Paresthesia during therapy was also recorded directly afterwards using a Likert-type scale and free text. None of the patients complained of “severe” or “very severe” paresthesia, and in the case of IPCT, no moderate paresthesia was reported. Consistency in the assessment of the paresthesia during therapy was found in 33/40 (82.5%) responses. Mild paresthesia during MLD compared to IPCT was found in 5/40 (12.5%) cases, and vice versa in 2/40 (5.0%) cases (Table 4).

The patients were asked whether they found the therapy pleasant directly after the respective intervention (see Table 5). Manual therapy was perceived as more pleasant than intermittent pneumatic compression, with a significance value of *p* = 0.013.

All 40 (100%) patients reported that they would recommend MLD, while 35 (87.5%) of the patients would recommend IPCT.

At the final follow-up, about two thirds (n = 27, 68%) of the patients stated that they preferred manual lymphatic drainage. Neither employment status (employed/unemployed) nor age (<67 years/≤67 years) influenced this decision (*p* = 1.000 and *p* = 0.316, respectively).

A total of 14 patients (35%) answered “yes” to the question “Could you imagine undergoing your lymphatic drainage therapy only with the automatic device?”.

## 4. Discussion

Chronic lymphedema is a highly relevant disease for the patients it affects and for the economy. Nevertheless, there is a gap in available reliable data regarding different forms of conservative therapy regimens for patients with chronic lymphedema [25]. IPCT is an established conservative therapy that is recommended as an additive therapy by the guidelines. To our knowledge, the efficacy of IPCT, especially in comparison to the standard therapy of MLD, has never previously been analyzed. This is an unsatisfactory study situation in the therapy of chronic lymphedema in general, and in MLD and IPCT in particular [21,22].

Female patients accounted for 80% of our study cohort. Two epidemiological studies on secondary lymphedema showed a similar gender distribution of 83% and 81% females, respectively [5,26]. In other studies, the mean age of the affected individuals was reported as 66.9 (±16.5), 59.7 (27–82), and 57.9 (34–76) years, respectively [25,26,27]. Our study participants were in a comparable age range. This shows that the analyzed cohort was representative of those suffering from secondary lymphedema.

The baseline volume had a significant effect on the volume difference. One could conclude that the lymphatic load could be mobilized from more voluminous lower legs. Volume measurements of the lower leg immediately after therapy did not reveal a significant difference between the two therapy methods. Neither the order in which treatment was administered, the lymphedema stage, nor the body mass index category had a significant effect. In contrast to the much more thoroughly studied lymphedema of the upper extremities, there are fewer data in the literature on volume reduction in the lower leg through a single therapeutic intervention. While a 55.7 mL or 1.47% reduction in the mean volume following MLD and 57.4 mL or 1.53% following IPCT might seem little, it should be mentioned that only the lower leg was considered, and the effect size is comparable to the results of other studies [14,18,28,29]. For a single intervention, it made no significant difference in terms of volume reduction whether the therapy was delivered by a therapist or by a device. Furthermore, only the affected leg was measured in this study. Whole-body fluid changes were not registered and might have an influence on the results.

With regard to the two-day effect, the initial volume had no significant influence, a period effect could not be demonstrated, and the volume difference of 0.39% ± 1.74 (MLD) and 0.27% ± 1.85 (IPCT) was very small and not of clinical relevance. The associated standard deviations are larger than the respective mean treatment effect and are in the positive as well as negative range. Thus, on the second postinterventional day, both volume decreases and increases can be observed with very different magnitudes. This suggests that a single intervention has no long-lasting effect on either MLD or IPCT. The selected washout period of 48 h was shown to be sufficient. Furthermore, these results are consistent with the clinical experience of both therapists and patients. It can be concluded that in many cases, a high-frequency decongestive therapy of up to daily applications can be useful. Intermittent pneumatic compression therapy with a device offers the possibility for the affected person to spontaneously adjust the frequency, duration, and intensity to the individual and the current lymphedema status. Ridner SH et al. were able to show good therapy adherence using home devices. They reported that 73% of noncancer-related lymphedema patients and 53% of cancer-related lymphedema patients who own an IPCT device use their device at least once a day. Only 7% of patients with lymphedema not secondary to cancer and 4% of patients with cancer-related lymphedema did not use the devices at all [30]. In addition, they were able to show that 95% of their subjects rated the effect of the devices as positive [30].

It is often stated by lymphatic therapists that the effect of manual lymphatic drainage is more long-lasting than that of other therapeutic methods due to the additional therapy of the central lymph node stations (inguinal, abdominal, and cervical). This cannot be confirmed with an interval of the measurement time points of two days in comparison with intermittent pneumatic compression.

The results of Uzkeser H. et al. showed that there was no advantage of additional treatment with IPCT in complex physical decongestive therapy [31]. However, these patients already received multiple therapies every day. Both Szuba A. et al. and Szolnoky G. et al. were able to show that there are synergistic effects in the combination of MLD and IPC [32,33].

There were no significant differences in the assessment of the two therapies by the subjects in the study presented, neither in the direct therapy effect, nor in the two-day-effect, pain, and discomfort. However, the subjects found MLD significantly more pleasant, although only five (12.5%) patients found IPCT unpleasant or very unpleasant. This, in turn, could be the reason why, despite the absence of differences in the subjective effectiveness, discomfort or pain, 68% of the subjects still preferred MLD to IPCT. IPCT was also less likely to be recommended to others (100% for MLD, 87.5% for IPCT therapy). The psychosocial interaction between the physiotherapist and the patient is probably of great importance. In cases where lymphedema is causing great suffering, patients can benefit enormously from positive, supportive communication with their therapists [7,8]; a device, of course, cannot provide this yet.

In the present study, only patients with secondary lymphedema were examined to ensure better comparability among subjects. No statement can be made about the effect of IPCT in primary lymphedema. In numerous studies, volumetric measurement is used for the objective evaluation of the therapeutic success of lymphatic drainage on the extremities. In reviews, it is one of the most important objectifiable and generally accepted criteria for the effectiveness of therapy [12].

There are various devices and cuffs used for IPCT. To address the question of a possible extension or implementation of the therapy in a home environment, we have chosen the “Lympha Press Mini—12-step system” (Version 201 ET, Mego Afek Ltd., Afek, Israel) with variable pressure control from 20 to 80 mmHg, which has been specifically optimized for home or self-therapy. The literature contains differentiated, sometimes contradictory information on the optimal pressure. Variable factors such as the degree of fibrosis of the lymphatic system, the variable subcutaneous tissue pressure, which is increased by up to 18 mmHg in the case of lymphedema, or simply the mass of soft tissue all have a significant influence on the optimal compression pressure. Data vary from 50 mmHg to 120 mmHg [4,18,23,29]. To date, however, there is no ‘best practice guideline’ [4,23,34]. To achieve the best possible comparability of results and considering the lack of sufficient data, we decided against an individualized pressure setting and in favor of a pressure setting of 80 mmHg, which was the same for all patients. This application pressure was tolerated by all patients for the entire duration of the application.

The inflation, compression, and deflation times with a cycle length of 30 s were dictated by the device. One study recommends inflation and deflation times of 50 s each [18]. Further studies, depending on the influencing factors, would be necessary here to optimize the compression cycles. To ensure the reproducibility of the intermittent pneumatic compression therapy, each subject was treated with the exact same treatment parameters.

Identical manual lymphatic drainage is inherently impossible between different therapists. Not even the same therapist can guarantee to apply the exact same drainage intensity and effectiveness in every therapy session. On the other hand, the individual patient–therapist relationship significantly influences the outcome of therapy [35]. A difference in the skills of different therapists is always present despite standardized and certified training. Therefore, it does not seem reasonable to work with a single therapist. MLD was performed by eight different certified therapists at the University Hospital Regensburg, all of whom were experienced in lymphatic drainage. This is to compensate for therapist-dependent variations in the measurement parameters of the subjective and objective data collection.

Surprisingly, no literature that examines the necessary or recommended duration of a therapy session for manual lymphatic drainage could be found. The guidelines also do not make any specific recommendations in this regard. For IPCT, the available data are sparse, have low-level evidence and are of moderate quality. These recommend application durations of 45–60 min [34]. Therefore, we followed commonly prescribed practices. Patients received exactly the same therapy duration of 45 min for both therapies.

One of the most important limitations of our study is the short observation period. The study took place during the COVID-19 pandemic. At that time, it was unclear how long the pandemic would last. This made it very difficult to recruit patients and conduct the study. A longer study period under these circumstances was unfortunately not possible. It is reported that long-term usage of IPCT may result in side effects (displacement of edema more proximally in the limb and genitalia or the development of a fibrosclerotic ring at the root of the extremity with exacerbated obstruction of lymph flow) [1]. Therefore, IPCT is currently recommended in combination with MLD [1]. Mendoza also found no differences in objective measurement between MLD alone, IPCT alone or both [36]. Further comparative studies covering a longer period of time are certainly necessary in order to obtain more meaningful results.

## 5. Conclusions

In summary, although both therapies are not perceived as significantly different in their effectiveness, patients preferred manual lymphatic drainage.

In this study, the subjective and objective therapy effects of manual lymphatic drainage and intermittent pneumatic compression for the therapy of secondary lymphedema of the lower extremity were investigated for the first time. No significant difference was found in the subjective and objective therapy effectiveness of the two methods in the 40 subjects studied. Intermittent pneumatic compression therapy is considered a comparable therapeutic procedure when properly indicated.

## Figures and Tables

**Table 1:** Results of the volumetric measurements immediately after therapy (immediate effect) and two days after therapy (two-day effect). m = mean volume difference (in mL).

	Therapy									
	MLD			IPCT						
	m	95% CI		m	95% CI		Mean Difference	95% CI		*p*
immediate effect	−57.01	−65.93	−48.09	−58.62	−67.59	−49.65	1.61	−9.99	13.22	0.780
two-day effect	−16.33	−35.86	3.19	−12.3	−34.24	9.64	−4.04	−37.91	29.84	0.811
	BMI categories									
	Obesity			non−obesity						
immediate effect	−51.96	−60.74	−43.19	−63.67	−75.37	−51.97	11.70	−3.88	27.29	0.137
two-day effect	−11.35	−26.66	3.97	−17.28	−37.68	3.11	5.94	−21.17	33.04	0.660
	lymphedema stage									
	I			II						
immediate effect	−53.99	−63.56	−44.41	−61.64	−70.96	−52.33	7.66	−5.45	20.76	0.245
two-day effect	−6.10	−22.79	10.6	−22.53	−38.77	−6.30	16.44	−6.33	39.20	0.152
	treatment order									
	MLD			IPCT						
immediate effect	−53.13	−62.71	−43.55	−62.5	−72.14	−52.87	9.37	−4.20	22.95	0.171
two-day effect	−12.27	−28.97	4.44	−16.36	−33.16	0.44	4.10	−19.49	27.68	0.727

**Table 2:** Results of subjective effectiveness immediately after therapy.

Subjective Effectiveness		IPCT			Total
		Moderate	Strong	Very Strong	
MLD	moderate	2	5	0	7
	strong	6	9	5	20
	very strong	1	8	4	13
Total		9	22	9	40

**Table 3:** Results of subjective long-term effectiveness in hours after therapy.

Subjective Long-Term Effectiveness in Hours		IPCT					Total
		<12	12–24	24–36	36–48	>48	
MLD	<12	4	1	0	1	0	6
	12–24	3	9	5	2	0	19
	24–36	0	5	3	4	1	13
	36–48	0	0	1	0	0	1
	>48	0	0	0	0	1	1
Total		7	15	9	7	2	40

**Table 4:** Subjective occurrence of paresthesia.

Paresthesia		ICPT			Total
		None	Mild	Moderate	
MLD	none	32	5	0	37
	mild	1	1	0	2
	moderate	0	1	0	1
Total		33	7	0	40

**Table 5:** Pleasantness of therapies.

Pleasant/Unpleasant		IPCT					Total
		Very Unpleasant	Slightly Unpleasant	Neither	Slightly Pleasant	Very Pleasant	
MLD	very unpleasant	0	0	0	0	1	1
	slightly unpleasant	0	0	0	1	0	1
	neither	0	0	0	0	0	0
	slightly pleasant	0	1	3	3	4	11
	very pleasant	1	3	4	7	12	27
Total		1	4	7	11	17	40

## Data Availability

Data can be made available on demand.
